# Ferroptosis in organ fibrosis: From mechanisms to therapeutic medicines

**DOI:** 10.2478/jtim-2023-0137

**Published:** 2024-03-21

**Authors:** Weijing Lai, Bo Wang, Rongshuang Huang, Chuyue Zhang, Ping Fu, Liang Ma

**Affiliations:** Department of Nephrology, Clinical Medical College and The First Affiliated Hospital of Chengdu Medical College, Chengdu, 610500, Sichuan Province, China; Department of Nephrology, Kidney Research Institute, West China Hospital of Sichuan University, Chengdu, 610041, Sichuan Province, China

**Keywords:** ferroptosis, organ fibrosis, fibrotic disease, anti-fibrotic therapy

## Abstract

Fibrosis occurs in many organs, and its sustained progress can lead to organ destruction and malfunction. Although numerous studies on organ fibrosis have been carried out, its underlying mechanism is largely unknown, and no ideal treatment is currently available. Ferroptosis is an iron-dependent process of programmed cell death that is characterized by lipid peroxidation. In the past decade, a growing body of evidence demonstrated the association between ferroptosis and fibrotic diseases, while targeting ferroptosis may serve as a potential therapeutic strategy. This review highlights recent advances in the crosstalk between ferroptosis and organ fibrosis, and discusses ferroptosis-targeted therapeutic approaches against fibrosis that are currently being explored.

## Introduction

Fibrosis is a pathological process defined as an excessive accumulation of extracellular matrix (ECM).^[1]^ Almost all vital organs can be affected by fibrosis, which manifests as increased fiber connective tissues and reduced parenchymal cells, whose sustained progress can lead to organ destruction and malfunction. Furthermore, almost half of all deaths were attributed to fibrosis in the developed world.^[2]^ Even though organ fibrosis has been studied for decades, its underlying mechanisms remain elusive, and no ideal treatment is currently available.

Ferroptosis is a novel form of programmed cell death, which is tightly regulated by intracellular signaling pathways, including but not limited to regulatory pathways for iron homeostasis and cysteine transport.^[3,4]^ The most characteristic feature of ferroptosis is the overwhelming iron-dependent accumulation of lethal lipid peroxides.^[5]^ Furthermore, many biomarkers involved in ferroptosis have been reported, among which cyclooxygenase-2 (COX2), Acyl-coenzyme A (CoA) synthetase long-chain family member 4 (ACSL4), and transferrin receptor 1 (TfR1) are upregulated, and glutathione peroxidase-4 (GPX4), solute carrier family 7 member 11 (SLC7A11), ferroptosis suppressor protein 1 (FSP1), and ferritin are down-regulated in ferroptotic cells.^[6,7,8,9,10]^ Cells undergoing ferroptosis lack classical alteration in the cell membrane, but rather they exhibit shrunken mitochondria, increased mitochondrial membrane density, reduced or absent mitochondrial ridge, and rupture of the mitochondrial outer membrane, while the nuclear size is normal without chromatin aggregation.^[8]^ Studies have confirmed that ferroptosis plays an important role in a variety of lesions in different organs.

**Figure 1:**
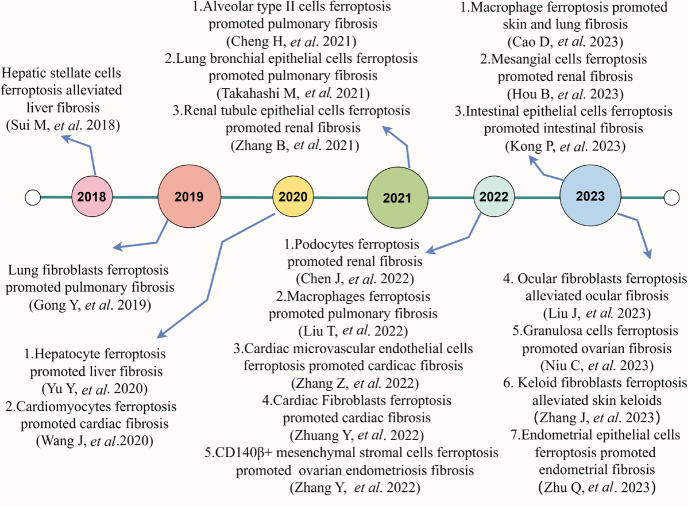
The timeline of cell ferroptosis in organ fibrosis.

In recent years, the role of ferroptosis in fibrotic processes has been attracting increasing interest (Figure 1). With the increasing volume of research on ferroptosis and fibrosis, comprehensive insights into how ferroptosis fundamentally and specifically affects fibrotic disease are critically needed. Therefore, in this review, we briefly summarized the current understanding of the ferroptosis drive and defense system in the process of fibrosis in multiple organs, as well as provided updated information on the potential of ferroptosis-targeting strategies for the treatment of organ fibrosis.

## Mechanisms of ferroptosis

The essence of ferroptosis is iron-dependent intracellular lipid peroxidation. Increasing evidence has suggested that factors responsible for lipid peroxidation and iron metabolism are involved in ferroptosis.^[3]^

### Iron metabolism

Although iron is vital for the biological function of a wide variety of cells, its aberrant accumulation could be as harmful as depletion.^[4]^ The key to the importance of iron in biological processes is its ability to reversibly cycle between its ferrous and ferric oxidation states to offer an e^-^ to oxygen, which could produce lipid peroxidation products (the Fenton reaction). Once iron is overloaded, it will accelerate the Fenton reaction, leading to lipid peroxide propagation and ferroptosis.^[11]^

Besides the non-enzymatic Fenton reaction, there is another iron-dependent pathway involved in lipid peroxidation, which is a highly selective and specific enzymatic lipoxygenase-controlled process.^[12]^ For example, lipoxygenases (LOXs), a class of iron-dependent dioxygenases, are a family of lipid peroxide enzymes that catalyze the oxidation of polyunsaturated fatty acids (PUFAs) and play an important role in the development of ferroptosis.^[13,14]^ Iron-containing enzymes with similar functions, including NADPH oxidases (NOXs) and cytochrome P450 (CYP).^[15,16]^

Given the critical role of iron in ferroptosis, the delicate regulation of iron metabolism is naturally vital for ferroptosis regulation. Iron homeostasis is strictly controlled by a series of proteins. Abnormal function of iron-regulated proteins leads to increased iron intake, or dysfunction of iron storage and elimination, resulting in an increased labile iron pool (LIP). The LIP is a significant source of oxidative radicals, serving as an initial element in ferroptosis.^[17]^ Thus, the iron-regulatory protein possessed the potential as a regulatory target against ferroptosis. To date, at least 19 major iron-regulated proteins have been reviewed to be involved in ferroptosis, including but not limited to CDGSH iron-sulfur domain 1/2, ferritin heavy chain 1, ferritin light chain, ferritin mitochondrial.^[18]^

Since iron overload is an essential feature of ferroptosis, some iron chelators have been proven to process inhibitory effects on ferroptosis, such as deferoxamine,^[19]^ deferiprone,^[20]^ dexrazoxane,^[21]^ and deferasirox.^[22]^ It is believed that more iron chelators will be confirmed to function as ferroptosis inhibitors in the future.

### Lipid peroxidation of polyunsaturated fatty acids

Lipid peroxidation is a crucial driving force of ferroptosis. PUFAs are straight-chain fatty acids with two or more double bonds. Due to the presence of diallyl matrix, PUFAs are vulnerable to hydrogen atom abstraction and are involved in the initiation of ferroptosis.^[13]^ The alkyl radicals produced by hydrogen extraction readily react with molecular oxygen to produce peroxyl radicals, which then react with other PUFAs to produce a chain reaction of lipid peroxidation.^[23]^ Previous studies indicated that PUFA could enhance lipid peroxidation and ferroptosis in MDA-MB-231 cells,^[24]^ whereas the unsaturated or monounsaturated fatty acids could not.^[13]^ The above further proved that PUFAs are important contributors to ferroptosis.

ACSL4, which is expressed in the endoplasmic reticulum and the mitochondrial outer membrane, can esterize CoA to free fatty acids and facilitate fatty acid oxidation.^[25]^ Specific inhibition of ACSL4 can significantly reduce lipid peroxidation and ferroptosis.^[26]^ However, the ferroptosis inhibitor Ferrostain-1 (Fer-1) and vitamin E could only lower the lipid peroxide content, but not the expression of ACSL4.^[27,28]^ These results suggested that ACSL4 catalyzes the initiation of lipid peroxidation and inhibition of ACSL4 could block ferroptosis from the source.

Lysophosphatidylcholine acyltransferase 3 (LPCAT3) is another enzyme that plays a key role in lipid peroxidation. As a main isoform of the four lysophatidylcholine acyltransferases in major metabolic tissues, LPCAT3 participates in the acyl transfer process of phosphatidylcholine remodeling in the mammalian cell membrane.^[29]^ The uncontrolled accumulation of arachidonoyl-(AA-) lipid hydroperoxides (OOH)-phosphatidylethanolamine (PE) was identified as the most important signal of ferroptosis.^[30]^ Specifically, LPCAT3 is an important enzyme that eternizes the AA-CoA to AA-PE,^[31]^ which is the basis for LOXs to convert AA-PE to AA-OOH-PE and promote ferroptosis.^[32]^ Thus, LPCAT3 is considered a well-deserved positive regulator for ferroptosis.^[33]^

### The xCT/GSH/GPX4 axis

The xCT is a Na^+^-independent cystine/glutamate antiporter on the cell membrane, which can import cystine and export glutamate.^[5]^ It consists of two main components: the transporter subunit SLC7A11 and the regulatory subunit solute carrier family 3 member 2 (SLC3A2).^[34]^ The antiporter plays a critical role in maintaining the intracellular production of glutathione (GSH), a major endogenous antioxidant.^[35]^ Inhibition of the xCT could decrease cystine uptake, leading to GSH consumption and cell ferroptosis.^[36]^ The tumor suppressor protein p53 was reported to enhance ferroptosis by inhibiting the xCT due to the transrepression of SLC7A11.^[37]^

GPX4 is a member of the GPX family and is the only GPX that functions as an enzyme to convert PL hydroperoxides to PL alcohol, thus repressing lipoxygenase-mediated lipid peroxidation.^[38]^ Inactivation or deletion of GPX4 leads to the accumulation of lipid peroxides, which is considered a lethal cause of ferroptosis. The expression or activity of GPX4 was widely known to be regulated by GSH and selenium. Either selenium deficiency or GSH deficiency is responsible for ferroptosis.^[34,39]^ Besides, SLC7A11-mediated cystine uptake was found to promote GPX4 protein synthesis via activating rapamycin complex 1 (mTORC1) in a GSH-independent way.^[40]^ Thus, the xCT/GSH/GPX4 axis is critical for defending against ferroptosis.

### The CoQH_2_ system

CoQ10 is a fat-soluble molecule with two forms: the complete oxidation state (CoQ) and the complete reduction state (CoQH_2_). CoQH_2_ is a radical-trapping antioxidant that can trap lipid peroxyl radicals and suppress lipid peroxidation and is considered a ferroptotic inhibitor.^[3]^

Dihydroorotate dehydrogenase (DHODH), an enzyme involved in pyrimidine synthesis, can reduce CoQ to CoQH_2_ and act as a compensator for GPX4 to suppress mitochondrial lipid peroxidation.^[9]^ Furthermore, FSP1, a NAD (P) H-dependent oxidoreductase, can also reduce CoQ to CoQH_2_ and exert its potent anti-ferroptosis activity in a glutathione-independent way.^[10,41]^ Similarly, GTP cyclohydrolase 1 (GCH1) can suppress ferroptosis through the GCH1-mediated production of CoQH_2._^[42,43]^ There are still some other CoQH_2_-producing enzymes, while their roles in ferroptosis regulation await further investigation.

Besides the above-described signaling molecules, nuclear factor erythroid-2 (NF-E2)-related factor 2 (Nrf2) has also been considered as an important ferroptosis defender that regulates both free-labile iron and lipid peroxidation.^[44]^ Furthermore, a recent study disclosed that phospholipidmodifying enzymes MBOAT1 and MBOAT2 as novel ferroptosis suppressors, which exerted effects through a GPX4-independent pathway by remodeling the cellular phospholipid profile.^[45]^

**Figure 2:**
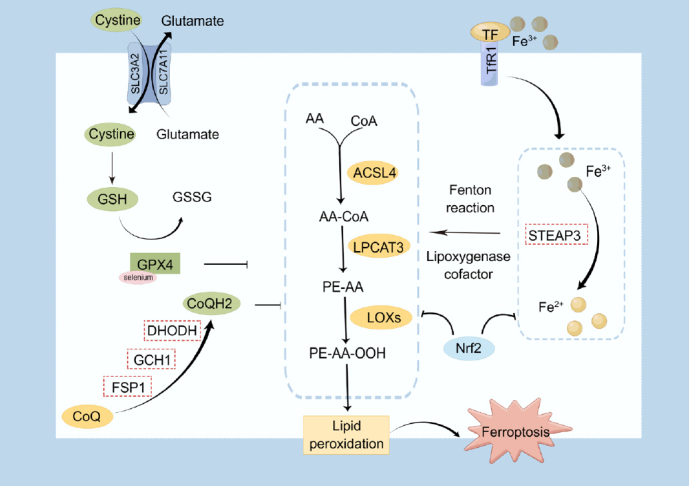
Overview of the ferroptosis mechanism. Ferroptosis occurs when the ferroptosis driving system (e.g., iron metabolism dysregulation, ACSL4 mediated lipid peroxidation) and ferroptosis defense system (e.g., xCT /GSH/GPX4 axis, CoQH_2_ system) are out of balance. GSH, glutathione; GSSG, Glutathione (oxidized); GPX4, glutathione peroxidase-4; AA, arachidonic acid; CoA, coenzyme A; ACSL4, acyl-CoA synthetase long-chain family member 4; LPCAT3, lysophosphatidylcholine acyltransferase 3; LOXs, lipoxygenases; PE, phosphatidylethanolamine; STEAP3, the six-transmembrane epithelial antigen of the prostate 3; TfR1, transferrin receptor 1; TF, transferrin; DHODH, dihydroorotate dehydrogenase; GCH1, GTP cyclohydrolase 1; FSP1, ferroptosis suppressor protein 1; Nrf2, nuclear factor erythroid-2 (NF-E2)-related factor 2. → presents the promote/activate and ⊥ presents the inhibitory/suppressive effects.

Overall, ferroptosis is tightly controlled by a complex web of signal regulations involved in iron metabolism and lipid peroxidation (Figure 2), and many avenues remain to be explored.

## Kidney fibrosis

Chronic kidney disease (CKD) is a growing global health problem that affects almost 10% of the world’s population and exerts a significant burden on public health.^[46]^ Kidney fibrosis is the common feature of CKD, which is characterized by excessive ECM deposition in the interstitial compartment, resulting in the destruction of normal kidney structure and eventual renal failure.^[47]^ Inhibiting the progression of renal fibrosis is essential to prevent renal function deterioration.

**Figure 3:**
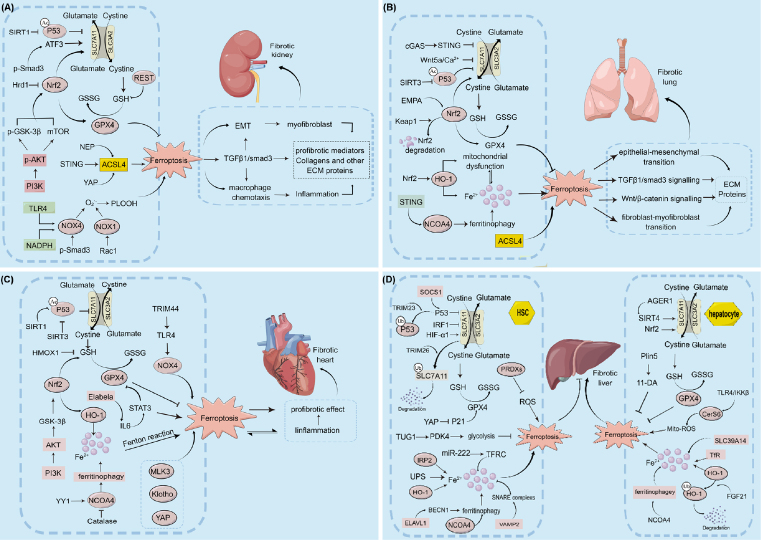
Ferroptosis in organ fibrosis (kidney, lung, heart, and liver). GSH, glutathione; GSSG, Glutathione (oxidized); GPX4, glutathione peroxidase-4; PI3K, phosphoinositide 3-kinase; AKT, protein kinase B; GSK-3β, glycogen synthase kinase-3β; Nrf2, nuclear factor erythroid 2-related factor 2; NADPH, nicotinamide adenine dinucleotide phosphate; NOX4, NADPH oxidase 4; Smad3, mothers against decapentaplegic homolog 3; HO-1, heme oxygenase 1; NCOA4, nuclear receptor coactivator 4; TGFβl, transforming growth factorβ1; REST, repressor element 1-silencing transcription factor; PLOOH, phospholipid hydroperoxides; MLK3, mixed lineage kinase 3; PI3K, phosphoinositide 3-kinase; AKT, protein kinase B; GSK-3β, glycogen synthase kinase-3β; cGAS, cyclic GMP-AMP synthase; UPS, ubiquitin-proteasome pathway; SNARE, soluble NSF attachment protein receptor; Ub, ubiquitination; suppressor of cytokine signaling 1; P53, tumor protein 53; HIFIαl, hypoxia-inducible factor-1α; EMPA, empagliflozin; IRP2, iron regulatory protein 2; TRIM, tripartite motif-containing protein; TRFC, transferrin Receptor; ELAVL1, ELAV-like RNA binding protein 1; BECN1, beclin 1; YY1, transcription factorYin Yang 1; HMOX1, heme oxygenase-1; VAMP2, vesicle-associated membrane protein 2; Rac1, ras-related C3 botulinum toxin substrate 1; STING, stimulator of interferon genes; YAP, yes-associated protein; TLR, toll-like receptor; TRIM, tripartite motif; PRDXs, peroxiredoxins; TfR, transferrin; FGF21, FGF21, fibroblast growth factor; SIRT, Sirtuin; SOCS1, suppressor of cytokine signaling 1; TUG1, taurine up-regulated gene 1; PDK4, pyruvate dehydrogenase kinase isozyme 4; Plin5, perilipin5; 11-DA, 11-Dodecenoic acid; CerS6, ceramide synthetase 6; IKKβ, I kappa B kinase beta; AGER1, AGE receptor 1; ROS, reactive oxygen species; EMT, epithelial-mesenchymal transition; ECM, extracellular matrix; NEP, neprilysin; ACSL4, acyl-CoA synthetase long-chain family member 4; Ac, acetylation; Ub, ubiquitination. → presents the promote/ activate and ⊥ presents the inhibitory/suppressive effects.

Recently, an increasing amount of data highlighted the correlation between ferroptosis and renal fibrosis, and a series of signaling pathways have been shown to participate in ferroptosis of renal tubular epithelial cells and promote renal fibrosis. For example, IRF1/ZNF350/ GPX4-mediated ferroptosis was disclosed to be responsible for the progression of chronic renal allograft interstitial fibrosis.^[48]^ Our studies confirmed that ACSL4-mediated ferroptosis was involved in UUO and adenine diet-induced kidney fibrosis.^[6,26]^ Other reported signaling pathways include Smad3/ATF3/SLC7A11 signaling,^[49]^ Akt/GSK-3p/Nrf2 signaling, ^[50]^ XBP1-Hrd1-Nrf2 signaling, ^[51]^ AKT/ mTOR/Nrf2 signaling,^[52]^ TLR4/Nox4 signaling ^[53]^ and so on (Figure 3A).

Mechanically, ferroptosis was involved in kidney fibrosis by promoting inflammation, epithelial-mesenchymal transition (EMT), etc. (Figure 3A). It was reported that the ferroptosis inhibitor Fer-1 could decrease MCP-1 production and reduce macrophage chemotaxis in NRK-52E cells, which were associated with progressive tubulointerstitial inflammation and fibrosis.^[54]^ Moreover, the ferroptosis inhibitor Deferoxamine (DFO) or Fer-1 could alleviate renal tubulointerstitial fibrosis by inhibiting the TGF-β1/ Smad3 pathway in UUO mice and 5/6 nephrectomy rats.^[55,56]^ The ferroptosis inhibitor Liproxstatin-1 (Lip-1) could attenuate renal fibrosis by reducing the activation of surrounding fibroblasts through inhibiting the paracrine of profibrotic factors in renal tubular epithelial cells.^[57]^ Furthermore, enrichment analysis of the core differential genes for ferroptosis in the normal population and hypertensive nephropathy samples found that ferroptosis may be involved in the occurrence and development of hypertensive nephropathy through the metabolism of branched-chain amino acid, retinol metabolism, biological processes such as organic amino acid metabolism and humoral immunity.^[58]^ However, much detail remains to be further clarified.

**Figure 4:**
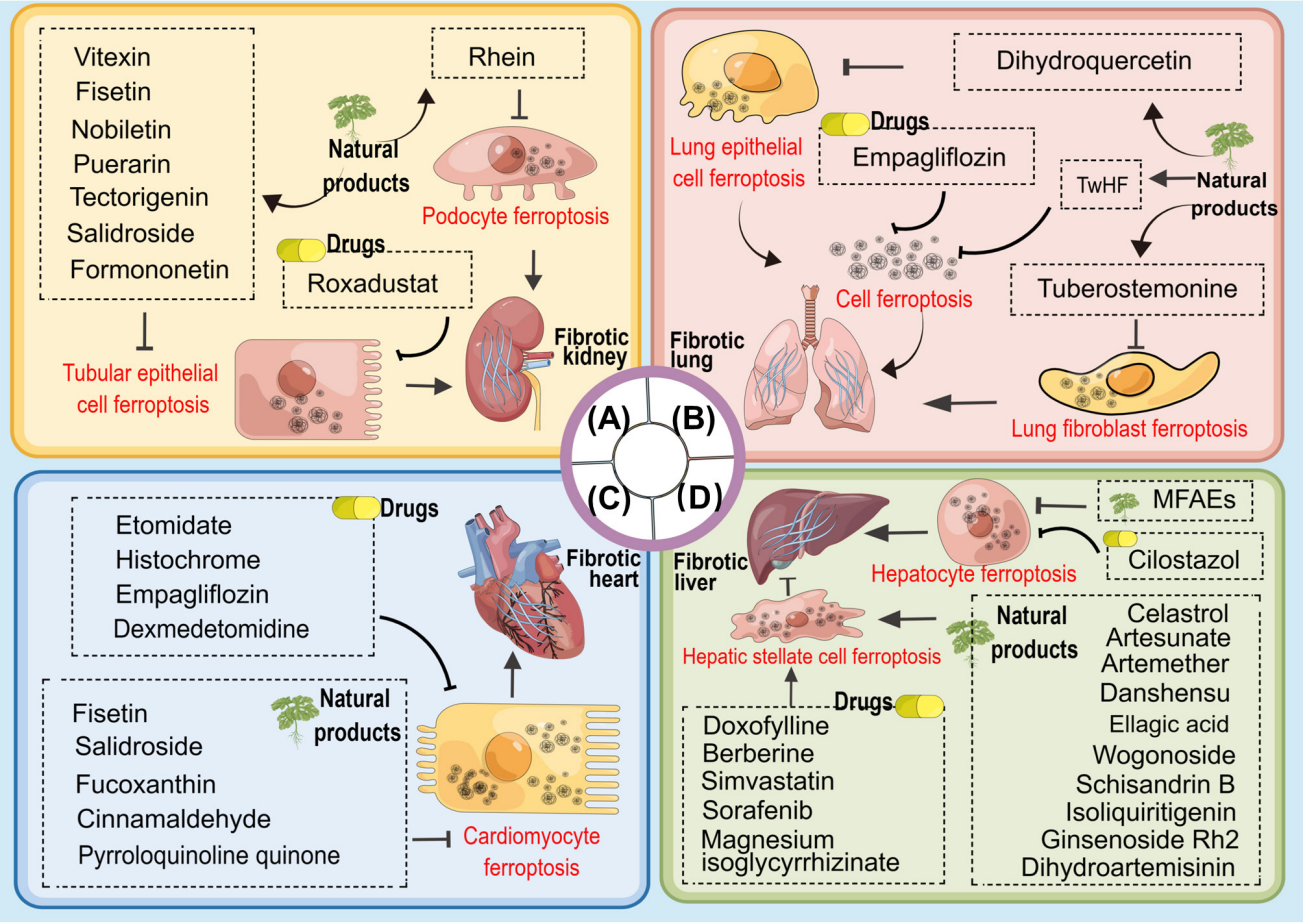
Natural products or drugs that play a therapeutic role in organ fibrosis through targeting ferroptosis. TwHF, tripterygium wilfordii Hook. f.; MFAEs, mori fructus aqueous extracts.

With the deepening of research, the therapeutic potential of targeting ferroptosis in fibrotic kidneys has been increasingly recognized (Figure 4A). In recent years, there has been an increased number of natural active ingredients found to possess anti-renal fibrotic effects, including vitexin,^[59]^ rhein,^[60]^ fisetin,^[6]^ puerarin,^[53]^ salidroside,^[61]^ formononetin,^[49]^ nobiletin,^[62]^ and tectorigenin.^[63]^ Furthermore, the marketed drug for renal anemia (roxadustat) was also found to exert its anti-fibrotic effects through targeting ferroptosis.^[50]^

## Pulmonary fibrosis

Pulmonary fibrosis is characterized primarily by inflammation and excessive deposition of ECM in the lungs, which causes irreversible structural damage and consequent pulmonary dysfunction.^[64]^ The most common form of pulmonary fibrosis is idiopathic pulmonary fibrosis (IPF), which affects 2.8 to 19 cases per 100, 000 people per year and with a median survival time of fewer than five years.^[64,65]^ Due to high incidence and poor prognosis, the mechanism of pulmonary fibrosis has long been focused on.

Recently, the association between ferroptosis and pulmonary fibrosis has been confirmed. Prussian blue staining showed that the number of cells positive for iron staining increased in lung tissues of patients with pulmonary fibrosis, while rare in healthy control.^[66]^ Furthermore, in bleomycin-induced fibrotic lung, cells positive for iron staining and total iron content increased significantly, and GPX4 expression levels decreased markedly.^[66,67]^ In the radiation-induced fibrotic lungs, subpleural collagen accumulations were significantly increased, accompanied by a significant decrease in GPX4 levels.^[68]^ Moreover, ferroptosis was also proved to be responsible for PM2.5-mediated pulmonary fibrosis and heavy metals-induced pulmonary fibrosis.^[69,70]^ To our knowledge, several ferroptosis pathways associated with pulmonary fibrosis have been revealed, including NCOA4-mediated ferritinophagy,^[71]^ miR-150–5p/ SLC38A1 signaling,^[72]^ Keap1/Nrf2 signaling,^[73]^ cGAS/ STING signaling,^[74]^ Nrf2/HO-1 signaling,^[75]^ Wnt5a/Ca^2+^ signaling,^[76]^ USP3/Sirt3/p53 signaling,^[77]^ EMPA/Nrf2/ SLC7A11 signaling,^[78]^ and ACSL4-dependent ferroptosis pathway (Figure 3B).

Nonetheless, there is limited data on how ferroptosis leads to pulmonary fibrosis (Figure 3B). In TGF-β1 stimulated pulmonary myofibroblasts, ferroptosis was found to promote fibrosis through fibroblast-to-myofibroblast differentiation, which has been confirmed as an important mechanism in pulmonary fibrosis.^[79]^ In A549 cells (human non-small cell lung cancer cells), the activation of ferroptosis was proved to be responsible for the EMT process, which was considered a contributor to pulmonary fibrosis.^[80]^ In the molybdenum and cadmium exposure-induced sheep model, the ferroptosis, due to the inhibition of the SLC7A11/GSH/GPX4 axis, was found to promote pulmonary fibrosis through the Cav-l/Wnt/β-catenin pathway.^[70]^ Moreover, the ferroptosis inhibitor Lip-1 could alleviate radiation-induced lung fibrosis through activating Nrf2 signaling and the subsequent down-regulation of TGF-β1signalling.^[68]^ However, more specific mechanisms need further exploration.

To date, emerging shreds of evidence indicated that targeting ferroptosis was effective in treating pulmonary fibrosis. In bleomycin-induced pulmonary fibrosis mice, the ferroptosis inhibitor DFO could decrease the intensity of lung inflammation and extracellular collagen deposition.^[66]^ In the radiation-induced pulmonary fibrosis mouse, ferroptosis inhibitor Lip-1 lowered the Szapiel and Ashcroft scores, and inhibited the collagen deposition in the lungs.^[68]^ In the graphene quantum dots-induced pulmonary fibrosis mouse, ferroptosis inhibitor Fer-1 also alleviated lung collagen deposition.^[81]^ Besides the abovementioned ferroptosis inhibitors, some natural active ingredients were also found to alleviate pulmonary fibrosis through inhibiting ferroptosis ^[75,82,83]^ (Figure 4B). Furthermore, empagliflozin, a sodium-glucose cotransporter 2 (SGLT2) inhibitor, was also found to ameliorate pulmonary fibrosis *via* targeting ferroptosis.^[78]^ Interestingly, in the bleomycin-induced mouse model, the pulmonary fibrosis could be ameliorated by DFO *via* transbronchial injection rather than oral administration or intraperitoneal injection,^[84]^ indicating that the route of administration should be considered when employing ferroptosis as a therapeutic target.

In addition to therapeutic targets, ferroptosis can also be used as a prognostic indicator for fibrotic pulmonary diseases. He Y *et al*. analyzed 293 ferroptosis-related genes and found that 19 of them were correlated with the prognosis of IPF, including NRAS, EMP1, MPO, SLC40AQ, MYC, ACO1, MYB, ACSF2, MIOX, ANGPTL4, GCH1, AIFM2, ATG7, FANCD2, PRKCA, MUC1, GABAPAPL1, and WIPI1.^[85]^ In the bronchoalveolar lavage fluid, except for MYB, GABARAPL1, ACO1, NRAS, GCH1, and MUC1, another 13 differentially expressed ferroptosis-related genes were also found to serve as prognostic biomarkers for IPF, including SRC, MT1G, SCD, CYBB, ALOX5, ZFP36, HIF1A, WIPI1, SLC40A1, ENPP2, LONP1, MAPK8, and HSF1, and further LASSO regression revealed that NRAS, ENPP2, MUC1, and ZFP36 are the risk factors, and ACO1 is a protective factor for IPF.^[86]^

## Cardiac fibrosis

Cardiovascular diseases are a leading cause of death globally. Cardiac fibrosis is a key factor for cardiac outcomes,^[87]^ and is characterized by the accumulation of ECM proteins that begin with cardiac fibroblast activation and lead to ventricular dysfunction and heart failure.^[88]^ The common process of cardiac fibrosis is that the death of cardiomyocytes promotes an inflammatory and fibrogenic response, which induces the formation of scar tissues.^[89]^

Programmed cell death has been established to play an important role in the progression of cardiac fibrosis, including ferroptosis.^[90]^ In fibrotic myocardial tissue of I/R induced rat model, ferroptosis characterized by the down-regulation of SLC7A11, GPX4, and GSH, and the up-regulation of COX2, ACSL4, PTGS4, and ROS were observed.^[91,92,93,94]^ Similarly, ferroptosis manifested as the decrease of GPX4 and the increase of total iron and lipid peroxidation were also presented in the cardiac tissues of rapid atrial pacing-induced atrial fibrillation beagle model, doxorubicin-induced cardiomyopathy mouse/rat model, adriamycin-induced cardiomyopathy rat model, transverse aortic constriction induced heart failure mouse model and angiotensin II-induced cardiac fibrosis, respectively.^[95,96,97,98,99,100,101,102,103]^ All the above results indicated the presence of ferroptosis in fibrotic heart diseases.

Many different ferroptosis signaling pathways have been reported to be involved in cardiac fibrosis (Figure 3C). In Ang II-induced cardiac fibrosis model, the xCT/ GPX4 pathway plays an important role in cardiomyocyte ferroptosis,^[104]^ and IL-6/STAT3/GPX4 signaling plays an important role in cardiac microvascular endothelial cell ferroptosis.^[105]^ In the STZ-induced DM mouse model, ferroptosis inhibition was proved to protect against diabetic-related cardiac fibrosis *via* enhancing the Nrf2/HO-1 pathway in cardiomyocytes.^[106]^ The Nrf2-dependent cardiomyocyte ferroptosis pathway was also established to be involved in cardiac fibrosis in the IRI model and the doxorubicin-induced cardiomyopathy model.^[107,108]^ Additionally, the PI3K/Akt pathway also plays an important role in triggering cardiac fibrosis through activating ferroptosis in ischemic injury condition.^[109]^ Recently, more and more ferroptosis signaling pathways have been found to participate in cardiac fibrosis, including TRIM44/TLR4/NOX4 signaling,^[110]^ SIRT1/ P53 signaling,^[111]^ SIRT3/P53 signaling,^[112]^ NCOA4 mediated ferritinophagy,^[113]^*et al*. However, the above mainly focused on mechanisms affecting ferroptosis in fibrotic cardiac diseases, the mechanism by which ferroptosis leads to cardiac fibrosis remains largely unknown. In hypertensive mice, although ferroptosis inhibition was found to alleviate fibrosis *via* downregulating profibrotic genes including fibronectin 1 (Fn1), connective tissue growth factor (CTGF), and TGF-β1,^[105]^ there is still much to explore.

Even so, the therapeutic effect of anti-ferroptosis on myocardial fibrosis has been focused on. In the IRI-induced rat model, the ferroptosis inhibitor Fer-1 could significantly attenuate myocardial fibrosis through the downregulation of iron levels and inhibition of the adverse effect of ROS.^[91]^ Furthermore, ferroptosis inhibition with Fer-1 was found to strikingly attenuate cardiac fibrosis in Ang II-induced cardiac fibrosis *in vivo* and *in vitro*.^[104, 105]^ Additionally, a series of natural products ^[96,99,100,107,114,115]^ and drugs ^[92,93,94,116]^ were found to alleviate cardiac fibrosis *via* inhibiting ferroptosis (Figure 4C).

## Liver fibrosis

Liver fibrosis is a wound-healing response to chronic liver injuries.^[117]^ To date, approximately 1.5 billion people are estimated to be suffering from fibrotic liver diseases worldwide, resulting in 2 million deaths per year.^[118]^ Therefore, it is imperative to delve into the molecular mechanisms to find new therapies.

As a vital organ involved in iron storage and metabolism, the liver is vulnerable to iron overload injury and is considered a preferred target organ for ferroptosis.^[119]^ A growing number of studies have shown the linkage between ferroptosis and liver fibrosis. Interestingly, in contrast to the previously described organs, ferroptosis can act as a double-edged sword in regulating liver fibrosis.^[120]^ Among the multiple progenitor cells in the liver, it was revealed that hepatocyte ferroptosis could promote the progression of liver fibrosis, while the activated hepatic stellate cells (HSCs) ferroptosis may attenuate liver fibrosis (Figure 3D).

Activated HSCs are the main source of collagen I and play a critical role in the progression of liver fibrosis,^[121]^ and their elimination is considered an effective anti-fibrotic strategy.^[122]^ Mechanisms linking HSC activation, liver ferroptosis, and liver fibrosis have gradually been revealed. For example, TfR overexpression was reported to trigger HSCs ferroptosis, and blocking miR-222 promoted activation of human HSCs cells and the following α-SMA and COL1A2 expression, so targeting miR-222/TfR signaling was considered to exert an anti-fibrotic effect in liver diseases.^[123]^ Moreover, the BRD7-P53-SLC25A28 axis was confirmed to mediate HSC ferroptosis *via* the mitochondrial iron metabolism pathway.^[124]^ Tripartite motif-containing protein 26, an E3 ubiquitin ligase, was shown to mitigate liver fibrosis by promoting HSCs ferroptosis *via* mediating SLC7A11 ubiquitination and degradation.^[125]^ Furthermore, sorafenib, a multiple kinase inhibitor, that exhibits an antifibrosis effect in the liver, was recently clarified to induce HSC ferroptosis through N6-methyladenosine (m6A) modification upregulation, autophagy activation, and HIF-1α/SLC7A11 signaling inhibition.^[126,127]^ Additionally, overexpression of the RNA-binding protein ZFP36 was found to protect against ferroptosis by triggering autophagy inactivation and blocking autophagic ferritin degradation in HSCs.^[128]^ Similarly, RNA-binding protein ELAVL1/HuR autophagydependent ferroptosis was found to be a protective mechanism for liver fibrosis.^[129]^ Additionally, magnesium isoglycyrrhizinate was shown to improve CCl4-induced liver fibrosis through HO-1-mediated HSC ferroptosis.^[130]^ Other reported pathways involved in HSCs ferroptosis include YAP/P21/GPX4 signaling, TUG1/PDK4 signaling, TRIM23/P53 signaling, *et al*. (Figure 3D). Based on these findings, some natural products and drugs were revealed to play an anti-fibrosis role in liver diseases through activating HSC ferroptosis ^[131,132,133,134,135,136,137,138,139,140,141,142,143]^ (Figure 4D).

In contrast to HSC, hepatocyte ferroptosis is considered a trigger of liver fibrosis (Figure 3D). For example, thymosin beta 4, which can alleviate inflammation and improve liver fibrosis, was verified to protect hepatocytes by inhibiting the GPX4-mediated ferroptosis pathway in human normal hepatocyte LO_2_ cell lines.^[144]^ Furthermore, constitutive activation of HO-1 was clarified to be responsible for ferroptosis of hepatocytes, and elevation of FGF21 could trigger HO-1 ubiquitination and subsequent degradation, therefore exerting protective effects in attenuating iron overload-induced liver injury and fibrosis.^[145]^ Additionally, the deletion of liver SLC39A14 expression could alleviate ferroptosis-mediated liver fibrosis induced by either a high-iron diet or CCl_4_ injections in hepatocyte-specific Trf-knockout mice.^[119]^ Moreover, AGER1 was found to ameliorate liver fibrosis by reversing EMT *via* inhibiting hepatocyte ferroptosis in nonalcoholic steatohepatitis with type 2 diabetes mellitus.^[146]^ Besides, liver fibrosis could also be alleviated by hepatocyte ferroptosis inhibition through targeting mitochondria-ROS and NCOA4-mediated ferritinophagy.^[147,148]^ Recently, mori fructus aqueous extracts were proven to attenuates liver fibrosis through inhibiting hepatocyte ferroptosis *via* the Nrf2 pathway.^[149]^ Cilostazol, an antiplatelet drug, was also found to alleviate liver fibrosis through inhibiting hepatocyte ferroptosis *via* the prevention of ectopic erythrophagocytosis.^[150]^ However, studies focused on the drugs targeting hepatocyte ferroptosis are still very limited.

Taken together, ferroptosis of HSCs and hepatocytes play opposite roles in liver fibrosis. Therefore, we had to consider whether systemic targeting of ferroptosis is appropriate. It has been reported that inhibiting xCT/ SLC7A11 induced ferroptosis of HSCs protected against acute liver fibrosis.^[35]^ However, the positive effects of inhibiting xCT/SLC7A11 in HSCs were overwhelmed by the negative effects of inhibiting xCT /SLC7A11 in hepatocytes in chronic liver injury.^[35]^ Thus, it is imperative to develop the targeted drugs.

## Ferroptosis and other fibrotic organs

The ovary and uterus are both important reproductive organs, and their fibrosis has a serious impact on female reproductive function and quality of life. Recently, granulosa cell ferroptosis was found to be responsible for chemotherapy-induced ovarian fibrosis,^[151]^ and spermidine was disclosed to alleviate ovarian fibrosis *via* regulating Nrf2/HO-1/GPX4 and Akt/FHC/ACSL4 pathway.^[152]^ Furthermore, ferroptosis was confirmed to be an important mechanism involved in endometrial fibrosis,^[153]^ and the humanin analogue was reported to improve endometrial fibrosis by inhibiting endometrial epithelial cell ferroptosis. Thus, targeting ferroptosis may be a promising strategy to combat female genital fibrosis.

Salivary glands are critical to maintaining oral health, their dysfunction can cause painful swelling, thick or purulent discharge, or dry mouth.^[154]^ Recently, the lipid and iron deposition were found to increase significantly in the submandibular gland tissue of post-menopause patients and ovariectomized animal models.^[155]^ Moreover, the submandibular gland fibrosis and saliva secretory disorder were aggravated after ovariectomy, and these lesions could be improved by the administration of ferroptosis inhibitors.^[156]^ Although these data suggested a contribution of ferroptosis to salivary gland fibrosis and dysfunction, the specific molecular mechanisms need to be further elucidated.

The intestine is one of the most inflammation-prone organs, and indeed one of the most susceptible organs to fibrosis. However, the pathogenesis of intestinal fibrosis is not completely confirmed, and no effective treatment strategy exists yet. Recently, ferroptosis of intestinal epithelial cells has been confirmed to be a promoting factor in the development of intestinal fibrosis.^[157]^ Specifically, STAT1-IRF1-ACSL4 axis-dependent ferroptosis was found to be an important mechanism in the process of interstitial fibrosis induced by radiation.^[157]^ Regretfully, current studies assessing the relationship between ferroptosis and interstitial fibrosis are very limited, and there remains a lot of room for further research.

Ocular fibrosis is characterized by pathological deposition of ECM in ocular tissues and eventually leads to blindness. Given the mechanistic similarity of fibrotic diseases, ferroptosisbased therapy has been conducted in ocular fibrosis. To date, artemisinin, an antimalarial traditional Chinese herb, has been proven to protect against ocular fibrosis through the induction of mitochondria-dependent ferroptosis in orbital fibroblasts.^[158]^ This finding may shed new light on ferroptosisbased therapy in treating ocular fibrosis.

Skin fibrosis is a kind of connective tissue lesion, which commonly includes keloid, hypertrophic scar, and scleroderma. With intensive studies on the pathogenesis of skin fibrosis, it was recently found that ferroptosis plays an important role in the process. As reported, ferroptosis induction in keloid fibroblasts was able to attenuate keloids.^[159]^ Furthermore, ACSL4 inhibition was found to prevent inflammatory macrophage ferroptosis and alleviate skin fibrosis in systemic sclerosis.^[160]^ Despite limited evidence, ferroptosis is undeniably one of the important mechanisms leading to skin fibrosis.

Besides the findings mentioned above, ferroptosis in intrahepatic bile duct epithelial cells was reported to be a potential target for the prevention and treatment of biliary fibrosis in fatty liver transplantation.^[161]^ Moreover, ferroptosis was found to be responsible for prostatic fibrosis in chronic Prostatitis.^[162]^ The role of ferroptosis in fibrotic conditions of other organs is still being explored.

## Conclusions and perspectives

Ferroptosis is emerging as a common factor involved in fibrotic diseases in different organs, including the kidney, lung, heart, liver, and so on. In general, ferroptosis has been considered a driver for most organ fibrosis, however, it seems that the effect of ferroptosis on organ fibrosis depends on the type of cell in which ferroptosis occurs, as the hepatocyte ferroptosis could promote liver fibrosis, and activated HSCs ferroptosis could alleviate liver fibrosis.

Complex molecular mechanisms are involved in the pathogenesis of ferroptosis-mediated organ fibrosis. Despite disease heterogeneity, the consensus mechanisms for ferroptosis-related organ fibrosis are the imbalance between the ferroptosis driving system (such as iron overload, lipid peroxidation) and the ferroptosis defense system (such as the xCT/GSH/GPX4 axis, CoQH_2_ system). Nevertheless, the signaling pathways are complex as interwoven networks and appear to vary in different organs and even in different cells of the same organ, and much remains to be explored.

Currently, targeting ferroptosis has been proven to be effective in alleviating organ fibrosis. In addition to common ferroptosis inhibitors and agonists, many natural products or drugs have also been identified. However, the pieces of evidence are currently limited to cell and animal experiments, clinical evidence is still lacking. Investigating the mechanism and developing targeted drugs remain important research topics, both now and future.
